# *BRCA1/2* Reversion Mutations in Patients Treated with Poly ADP-Ribose Polymerase (PARP) Inhibitors or Platinum Agents

**DOI:** 10.3390/medicina58121818

**Published:** 2022-12-10

**Authors:** Sourat Darabi, David R. Braxton, Joanne Xiu, Benedito A. Carneiro, Jeff Swensen, Emmanuel S. Antonarakis, Stephen V. Liu, Rana R. McKay, David Spetzler, Wafik S. El-Deiry, Michael J. Demeure

**Affiliations:** 1 Hoag Family Cancer Institute, Newport Beach, CA 92663, USA; 2 Caris Life Sciences, Phoenix, AZ 85040, USA; 3 Legorreta Cancer Center, Brown University, Providence, RI 02912, USA; 4 University of Minnesota Masonic Cancer Center, Minneapolis, MN 55455, USA; 5 Georgetown University, Washington, DC 20057, USA; 6 University of California San Diego Health, La Jolla, CA 92093, USA; 7 Translational Genomics Research Institute, Phoenix, AZ 85004, USA

**Keywords:** *BRCA1/2*, reversion mutations, PARP inhibitors, resistance mechanisms, platinum-based therapy

## Abstract

*Background*: Reversion mutations in *BRCA1/2*, resulting in restoration of the open reading frame, have been identified as a mechanism of resistance to platinum-based chemotherapy or PARP inhibition. We sought to explore the incidence of *BRCA1/2* reversion mutations in different tumor types. *Methods*: We retrospectively analyzed molecular profiling results from primary and/or metastatic tumor samples submitted by multiple institutions. The samples underwent DNA and RNA sequencing at a CLIA/CAP-certified clinical lab. Reversion mutations were called only in patients whose available clinical records showed the use of PARP inhibitors or platinum agents prior to tumor profiling. *Results*: Reversion mutations were identified in 75 of 247,926 samples profiled across all tumor types. Among patients carrying pathogenic or likely pathogenic *BRCA1/2* mutations, reversion mutations in *BRCA1/2* genes were seen in ovarian cancer (OC) (30/3424), breast cancer (BC) (27/1460), endometrial cancer (4/564), pancreatic cancer (2/340), cholangiocarcinoma (2/178), prostate cancer (5/461), cervical cancer (1/117), cancer of unknown primary (1/244), bladder cancer (1/300), malignant pleural mesothelioma (1/10), and a neuroendocrine tumor of the prostate. We identified 22 reversion mutations in *BRCA1* and 8 in *BRCA2* in OC. In BC, we detected 6 reversion mutations in *BRCA1* and 21 in *BRCA2.* We compared molecular profile results of 14 high-grade serous ovarian cancers (HGSOC) with reversion mutations against 87 control HGSOC with pathogenic *BRCA1/2* mutations without reversion mutations. Tumors with reversion mutations trended to have had lower ER expression (25% vs. 64%, *p* = 0.024, q = 0.82) and higher *KDM6A* mutation rate (15% vs. 0, *p* = 0.016, q = 0.82). *Conclusions*: We present one of the largest datasets reporting reversion mutations in *BRCA1/2* genes across various tumor types. These reversion mutations were rare; this may be because some patients may not have had repeat profiling post-treatment. Repeat tumor profiling at times of treatment resistance can help inform therapy selection in the refractory disease setting.

## 1. Introduction

Advances in genomic technologies in next-generation sequencing (NGS) resulting in rapid and low-cost genomic testing paved the way for more molecular testing of tumors and subsequent research toward developing targeted treatments. NGS technologies can detect driver mutations in cancer, including in the genes involved in the homologous recombination (HR) DNA repair pathway, such as *BRCA1, BRCA2, PALB2, RAD51C, RAD51D, ATM, CHEK2, BARD1, CDK12*, and others. Defects in the HR pathway are common in triple-negative breast cancer, high-grade serous ovarian cancer, and castration-resistant metastatic prostate cancer [1].

Tumors with HR deficiency are sensitive to platinum-based chemotherapy or PARP inhibitors due to synthetic lethality [2], however, primary and acquired resistance to these therapies has been reported [2,3]. In different tumor types, such as ovarian, prostate, pancreatic, and breast cancer, the development of *BRCA 1/2* reversion mutations has been reported as a resistance mechanism [3,4,5,6]. Some patients may have tumors with more than one reversion mutation [7,8]. In a large cohort of the Chinese cancer population, the frequency of reversion mutations in pan-cancer unselected patients was 1.7%, with the most events reported in *BRCA1, BRCA2*, and *PALB2* genes [2]. Another group of researchers analyzed over 300 reversion mutations in *BRCA1/2* genes to determine whether there is an association between specific regions in these genes and the risk of reversion mutations. They reported “hot spot” and “desert” regions in these genes. In addition, missense and splice site variants are less likely to show reversion mutations compared with truncating mutations in *BRCA1/2* genes [1].

Herein, we analyzed molecular tumor profiling results in a pan-cancer patient set who had testing performed at a CLIA/CAP certified laboratory and report the number of identified reversion mutations in patients who had been treated with either platinum-based therapy or PARP inhibition.

## 2. Materials and Methods

We retrospectively analyzed molecular sequence data from tumor samples for which DNA and RNA were available.

### 2.1. Next-Generation Sequencing (NGS)

NGS was performed on genomic DNA isolated from formalin-fixed paraffin-embedded (FFPE) tumor samples using the NextSeq or NovaSeq 6000 platforms (Illumina, Inc., San Diego, CA, USA). For NextSeq sequenced tumors, a custom-designed SureSelect XT assay was used to enrich 592 whole-gene targets (Agilent Technologies, Santa Clara, CA, USA). For NovaSeq sequenced tumors, more than 700 clinically relevant genes at high coverage and high read-depth was used, along with another panel designed to enrich for an additional >20,000 genes at a lower depth (Supplemental Table S2). All variants were detected with >99% confidence based on allele frequency and amplicon coverage, with an average sequencing depth of coverage of >500 and an analytic sensitivity of 5%. Prior to molecular testing, tumor enrichment was achieved by harvesting targeted tissue using manual microdissection techniques. Genetic variants identified were interpreted by board-certified molecular geneticists and categorized as ‘pathogenic’ ‘likely pathogenic’ ‘variant of unknown significance’ ‘likely benign’ or ‘benign’ according to the American College of Medical Genetics and Genomics (ACMG) standards. When assessing mutation frequencies of individual genes, ’pathogenic’ and ‘likely pathogenic’ were counted as mutations. The copy number alteration (CNA) of each exon is determined by calculating the average depth of the sample along with the sequencing depth of each exon and comparing this calculated result to a pre-calibrated value. Reversion mutations were identified by a board-certified molecular geneticist and called only if the patient had been treated with a PARP inhibition or a platinum agent.

Tumor mutational burden (TMB) was measured by counting all non-synonymous missense, nonsense, in-frame insertion/deletion and frameshift mutations found per tumor that had not been previously described as germline alterations in dbSNP151, Genome Aggregation Database (gnomAD) databases or benign variants identified by Caris geneticists. A cutoff point of ≥10 mutations per megabase (MB) was used based on the KEYNOTE-158 pembrolizumab trial [9], which showed that patients with a TMB of ≥10 mt/MB across several tumor types had higher response rates than patients with a TMB of <10 mt/MB. Caris Life Sciences is a participant in the Friends of Cancer Research TMB Harmonization Project [10].

### 2.2. Whole Transcriptome Sequencing

Gene fusion detection was performed on mRNA isolated from an FFPE tumor sample using the Illumina NovaSeq platform (Illumina, Inc., San Diego, CA, USA) and Agilent SureSelect Human All Exon V7 bait panel (Agilent Technologies, Santa Clara, CA, USA). FFPE specimens underwent pathology review to diagnose percent tumor content and tumor size; a minimum of 10% of tumor content in the area for microdissection was required to enable enrichment and extraction of tumor-specific RNA. Qiagen RNA FFPE tissue extraction kit was used for extraction, and the RNA quality and quantity was determined using the Agilent TapeStation. Biotinylated RNA baits were hybridized to the synthesized and purified cDNA targets and the bait-target complexes were amplified in a post capture PCR reaction. The resultant libraries were quantified, normalized and the pooled libraries were denatured, diluted and sequenced; the reference genome used was GRCh37/hg19 and analytical validation of this test demonstrated ≥97% Positive Percent Agreement (PPA), ≥99% Negative Percent Agreement (NPA) and ≥99% Overall Percent Agreement (OPA) with a validated comparator method.

### 2.3. CODEai

Real-world overall survival (rwOS) information was obtained from insurance claims data and calculated from either first of treatment time to last of treatment time (TOT). Kaplan–Meier estimates were calculated for molecularly defined patient cohorts. Significance was determined as *p* values of <0.05.

### 2.4. Statistics

The comparison of the molecular alterations between the two groups was performed using ChiSquareChi-square and Fisher exact whenever appropriate. Benja-mini-Hochberg method was used for calculating adjusted *p* values (i.e., q values), and a q < 0.05 was regarded as statistically significant; *p* < 0.05 but q > 0.05 was regarded as a trending difference.

## 3. Results

We analyzed tumor profiling results from all 247, 926 solid tumors profiled from primary or metastatic samples submitted due to clinical indications. Reversion mutations were identified and reported in *BRCA1* or *BRCA2* genes by a board-certified medical geneticist. These mutations are only called if the patient’s medical record submitted to Caris Life Sciences to accompany the request for tumor profiling test showed a record of a prior platinum agent or PARP inhibition use. Among the 247,926 tumors, we identified reversion mutations in 75 tumor samples: 32 with *BRCA1* and 43 with *BRCA2* reversion mutations. We detected the highest number of reversion mutations in ovarian cancer (n = 30), followed by breast cancer (n = 27) (Table 1, Supplemental Table S1). More cases with *BRCA1* reversion mutations were reported in ovarian cancer than *BRCA2*. In contrast, more *BRCA2* reversion mutations were detected in breast cancer than *BRCA1.* Reversion mutations were reported in 30 out of 3424 tumors with *BRCA1/2* mutations in ovarian cancer and 27 out of 1460 tumors with mutations in breast cancer. The remaining reversion mutations were reported in endometrial cancer (4/564), pancreatic cancer (2/340), cholangiocarcinoma (2/178), prostate cancer (5/461), cervical cancer (1/117), cancer of unknown primary (1/244), and a neuroendocrine tumor of the prostate, as well as in a case of malignant pleural mesothelioma (1/10) and a case of bladder cancer (1/300).

**Figure 1:**
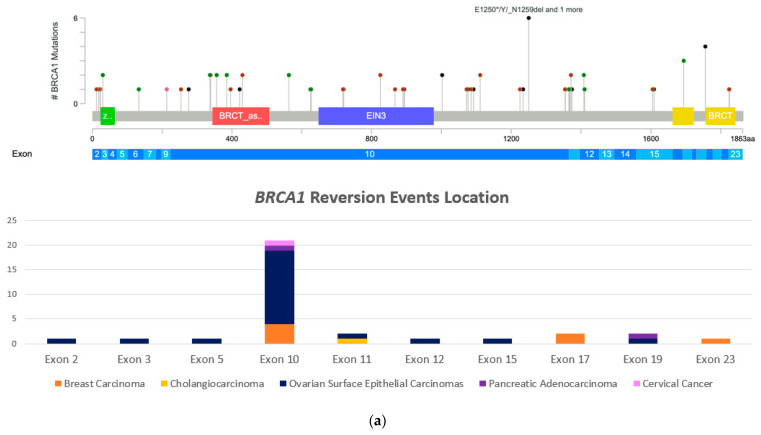

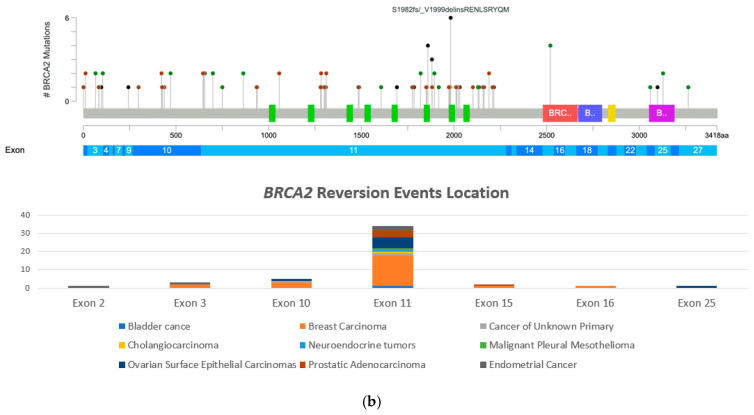
Lollipop plot of reported reversion mutations. (**a**) Reversion mutations in *BRCA1* gene with corresponding affected exons and by tumor type. (**b**) *BRCA2* reversion mutations with corresponding affected exons by tumor type (MutationMapper by cBioPortal: https://www.cbioportal.org/mutation_mapper, accessed on 7 November 2022).

The distribution of mutations on *BRCA1/2* genes is shown in the lollipop plot in Figure 1. The Mutation Mapper tool was used to illustrate the mutation locations. In addition, we show mutation locations by cancer type in each gene. Affected exons are also noted in Figure 1a,b. We observed most *BRCA1* reversion mutations were in exon 10 (18 out of 28 *BRCA1* reversion mutations) and most *BRCA2* reversion mutations were in exon 11 (16 out of 26 *BRCA2* reversion mutations). Additional patients are needed to further analyze the significance of this observation as these exons are large and this may be the explanation for the clustering of the mutations in these exons.

**Figure 2:**
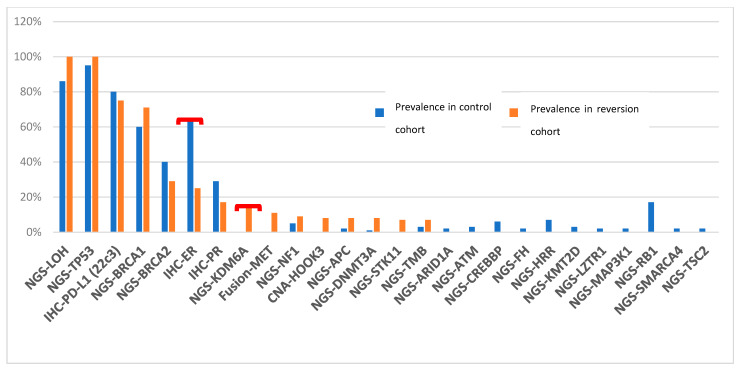
Comparing molecular profiling results of ovarian high-grade serous *BRCA1/2* mutant tumors with or without reversions (Red brackets denote *p* < 0.05). The most prevalent altered biomarkers are shown.

In addition, the molecular and genetic features of a cohort of 87 patients with high-grade serous ovarian cancer (HGSOC) harboring *BRCA1*/2 mutations without reversion mutations were compared with those of 14 patients with HGSOC and reversion mutations. Tumors with reversion mutations in *BRCA1/2* genes trended to have lower ER expression identified by immunohistochemistry (IHC) (25% vs. 64%, *p* = 0.024, q = 0.82) and higher *KDM6A* mutation rate (15% vs. 0, *p* = 0.016, q = 0.82). We did not detect any *RB1* mutations in cases with reversion mutations (Figure 2).

Furthermore, from insurance claims data, we were only able to collect more detailed clinical history information for 29 of the patients with reversion mutations, 17 with *BRCA1* and 12 with *BRCA2* mutations. Seven patients had prior treatment with a platinum-based chemotherapy, cisplatin or carboplatin. Seven of them were treated with PARP inhibitors, rucaparib or olaparib, and seven patients had both platinum chemotherapy and PARP inhibition. Notably, five patients were treated with carboplatin (n = 2, ovarian), olaparib (n = 1, breast), or both agents (n = 2, ovarian and prostate) after the detection of reversion mutations.

An article reporting four cases showed that inactivating *TP53* mutations, either de novo or pre-existing in *BRCA1/2* mutated ovarian/breast tumors, may be associated with resistance to olaparib [11]. Similar findings have been observed in prostate cancer [12]. As the presence of a *TP53* mutation at the time of diagnosis may promote chemotherapy resistance, we evaluated *TP53* mutations in the ovarian cancer cohort. Amongst patients with HGSOC, *TP53* mutation rates were similar in reversion mutations and control groups (100% vs. 95%). In patients with reversion mutations, 7 of the 14 (50%) *TP53* mutations were gain-of-function (GOF), while only 19 of 84 (23%) *TP53* mutations in the control group were GOF (*p* = 0.048), suggesting that *BRCA* reversion events may be a more plausible resistance mechanism in the context of *TP53* GOF mutant ovarian cancer.

## 4. Discussion and Conclusions

Advances in genomic technologies have guided the development of precision-oncology treatments. Deficient HR is caused by mutations in several genes in this pathway, including *BRCA1/2*. There are FDA-approved treatments as well as clinical trials targeting cancers with these mutations. DNA-damaging agents or PARP inhibitors are approved in certain tumor types with specific molecular sub-types. As more drugs are approved by the FDA, we have begun to observe resistance mechanisms in different tumor types [13]. Herein, we report reversion mutations in 75 cancers of various histologies. The majority of cases were in ovarian and breast cancer. Most of these reversion mutations were observed in *BRCA1* gene involving exon 10 (the largest exon in *BRCA1* gene) and in *BRCA2* involving exon 11.

We reported herein additional reversion mutations in other tumor types including endometrial cancer, pancreatic cancer, prostate cancer, cervical cancer, cholangiocarcinoma, cancer of unknown primary, and a neuroendocrine tumor. This data shows that while these mutations are seen in breast and ovarian cancer, there are also reported in patients with other cancer types. Reversion mutations are well-documented in breast, ovarian, and prostate cancers but rarely in pancreatic cancers and other tumors. Pancreatic cancer is often treated with platinum-containing chemotherapy combinations and only recently, maintenance therapy with PARP inhibition has been studied [14]. Our data highlights the prevalence and possible importance of reversion mutations in other tumors, but further investigation is required. These patients may be eligible for alternative treatments based on their molecular profiling results including immunotherapy, or clinical trials to overcome the resistance.

We can conclude, however, regardless of tumor type, it is important, to repeat molecular profiling by either tumor sequencing or liquid biopsy if a patient’s tumor progresses on platinum-based therapy or PARP inhibition. In our patient population, 6 patients had breast cancers that also had high TMB, and 4 patients had other cancers exhibiting high TMB. Immunotherapy may be an option for patients with high TMB as an alternative [15]. Some investigators have postulated that a combination of pembrolizumab and PARPi may have efficacy [16]. It is not clear, however, whether the combination of these agents would have benefit in the presence of a reversion mutation.

Microhomology-mediated end-joining (MMEJ) repair was reported in 58.3% of reversion deletions in the cohort of patients with *BRCA1/2* deletions [2]. This may be the mechanism that is involved in resistance. Further investigation of raw sequencing data to analyze microhomology is planned. Some early studies have shown a drug called POLƟ inhibitors can prevent MMEJ in combination with PARP inhibition or platinum-based therapy could delay the resistance [17]. A subset of patients may also benefit from a combination of WEE1 inhibitors and PARP inhibitors or ATR inhibitors [18].

There are limitations to our study. As we took advantage of a large cohort of real-world database housing clinical samples submitted for tumor profiling as part of standard-of-care, we did not have detailed outcome data for all patients. Our observations suggest that in clinical practice, tailoring patient’s PARP inhibitor/platinum treatment based on *BRCA* mutation status is not fully implemented and that additional clinical studies on the clinical impact of *BRCA* reversion mutations are still needed to further awareness in the oncology community. Collecting additional patient clinical data would allow for further description of these reversion mutations for molecular and clinical behavior.

Another limitation of our study is that primary tumors were not available to compare with the samples collected at the time of resistance, therefore, it is unclear if subclones of the cancer cells harboring reversion mutations were present at the time of diagnosis and were amplified under the selection pressure of the DNA-damaging agents, or if the reversion mutations were an acquired event at the time of resistance. It would be helpful to have paired pre- and post-treatment samples to have a better understanding of the incidence of these reversion mutations.

We conclude that while *BRCA1/2* reversion mutations are rare events, repeat molecular tumor profiling at the time of treatment resistance may help guide therapy selection in the refractory disease setting. This includes discontinuation of treatment with platinum-based therapy or PARP inhibition. Patients may also have options for clinical trials on new emerging treatments.

## Figures and Tables

**Table 1:** The number of detected *BRCA1/2* mutations and total of reversion mutations (RM) in different cancer types in our cohort of 247,926tumor samples.

	*BRCA1* RM (N)	*BRCA2* RM (N)	*BRCA1* Pathogenic Counts	*BRCA2* Pathogenic Counts	Total Result Counts
Ovarian Cancer	22	8	2076	1348	25,499
Breast Cancer	6	21	610	850	21,157
Prostate Cancer	0	5	57	404	7796
Endometrial Cancer	0	4	176	388	9521
Pancreatic Cancer	2	0	115	329	11,163
Cholangiocarcinoma	1	1	50	128	5652
Bladder Cancer	0	1	100	200	6552
Cervical Cancer	1	0	43	74	3267
Cancer of Unknown Primary	0	1	93	151	6283
Neuroendocrine tumor	0	1	30	66	3786
Malignant Pleural Mesothelioma	0	1	2	8	463
Total	32	43	3352	3946	101,139

## Data Availability

The CARIS datasets generated during and/or analyzed during the current study are available from the corresponding author on reasonable request. The deidentified sequencing data are owned by Caris Life Sciences. Qualified researchers can apply for access to these summarized data by contacting Joanne Xiu, PhD and signing a data usage agreement.

## Supplementary Materials

The following supporting information can be downloaded at: https://www.mdpi.com/article/10.3390/medicina58121818/s1, Table S1: *BRCA1* and *BRCA2* reversion mutation details. Table S2 title: Gene lists on the 592-gene panel and WES panel with high coverage.
