# Supplementary material for: BRCA1/2 Reversion Mutations in Patients Treated with Poly ADP-Ribose Polymerase (PARP) Inhibitors or Platinum Agents
Source: Medicina (Kaunas). 2022 Dec 10;58(12):1818. doi: 10.3390/medicina58121818 (PMC9785940; doi:10.3390/medicina58121818)
Supplement: Supplementary file 1 [file medicina-58-01818-s001.zip › medicina-2060065-supplementary.pdf]

Table S1. *BRCA1* and *BRCA2* reversion mutation details.

	Cancer Type	*BRCA1* mutation 1				*BRCA1* mutation 2				*BRCA1* mutation 3			
		p. changes	c. changes	Exon	[illegible]	p. changes	c. changes	Exon	VAF	p. changes	c. changes	Exon	VAF
**1**	**Breast**	E1694Y	c.5080_5082delinsTAC	17	[illegible]	E1694L	c.5080_5081delinsTT	17	8	E1694*	c.5080G>T	17	58
**2**	**Breast**	K893fs	c.2679_2682delGAAA	10	[illegible]	Q867_K893delinsMCNILCPLWVL	c.2598_2679delins34	10	22				
**3**	**Breast**	Q563Y	c.1687_1689delinsTAC	10	[illegible]	Q563*	c.1687C>T	10	69				
**4**	**Breast**	R1076fs	c.3227delG	10	[illegible]	L1072_R1076delinsQ	c.3215_3227delinsA	10	25				
**5**	**Breast**	V627fs	c.1874_1877dupTAGT	10	[illegible]	L625Y	c.1873_1875delinsTAT	10	38				
**6**	**Breast**	G1825fs	c.5474_5481del8	23	[illegible]	I1824_Q1826del	c.5472_5480del9	23	21				
**7**	**Cerivcal**	L718_E720del	c.2152_2160del9	10	[illegible]	E720fs	c.2157dupA	10	29				
**8**	**Cholangiocarcinoma**	S1374fs	c.4120_4121delAG	11	[illegible]	A1368_E1373delinsQHLGV	c.4101_4118delins15	11	16				
**9**	**Ovarian**	G1366S	c.4096G>A	10	[illegible]	E1250Y	c.3748_3750delinsTAT	10	19	E1250*	c.3748G>T	10	54
**10**	**Ovarian**	L30Y	c.89_90delinsAC	3	[illegible]	L30*	c.89T>A	3	50				
**11**	**Ovarian**	c.213-13_213-12delinsTG	c.213-13_213-12delinsTG	5	[illegible]	c.213-12A>G	c.213-12A>G	5	69				
**12**	**Ovarian**	W385L	c.1154_1155delinsTA	10	[illegible]	W385*	c.1155G>A	10	56				
**13**	**Ovarian**	C1225_R1243del	c.3673_3729del57	10	[illegible]	V1234fs	c.3700_3704delGTAAA	10	47				
**14**	**Ovarian**	Q1111fs	c.3331_3334delCAAG	10	[illegible]	Q1111del	c.3330_3332delGCA	10	42				
**15**	**Ovarian**	Q1408L	c.4222_4223delinsTT	12	[illegible]	Q1408*	c.4222C>T	12	64				
**16**	**Ovarian**	R1085_Q1096del	c.3255_3290del36	10	[illegible]	E1092fs	c.3273delT	10	45				
**17**	**Ovarian**	L22_I26del	c.65_79del15	2	[illegible]	E23fs	c.68_69delAG	2	61				
**18**	**Ovarian**	D825_I845delinsEQKALSIHWDMKLTT	c.2475_2534delins42	10	[illegible]	D825fs	c.2475delC	10	49				
**19**	**Ovarian**	Q1756_R1758delinsPGQ	c.5267_5273delins7	19	[illegible]	Q1756fs	c.5266dupC	19	68				
**20**	**Ovarian**	N1354_E1357del	c.4060_4071del12	10	[illegible]	N1355fs	c.4065_4068delTCAA	10	52				
**21**	**Ovarian**	Q356*	c.1066C>T	10	[illegible]	Q356E	c.1066C>G	10	30				
**22**	**Ovarian**	E1250_N1259del	c.3748_3777del30	10	[illegible]	E1250*	c.3748G>T	10	19				
**23**	**Ovarian**	D396_S425del	c.1186_1275del90	10	[illegible]	Y422*	c.1266T>G	10	49				
**24**	**Ovarian**	E1609fs	c.4826_4837delinsG	15	[illegible]	L1605_S1613delinsG	c.4812_4837delinsGG	15	25				
**25**	**Ovarian**	E337K	c.1009G>A	10	[illegible]	E337fs	c.1008delA	10	45				
**26**	**Ovarian**	K894fs	c.2681_2682delAA	10	[illegible]	G890_K894delinsR	c.2667_2681delinsAAG	10	21				
**27**	**Ovarian**	G1371_S1383del	c.4110_4148del39	11	[illegible]	G1371fs	c.4110_4111delTG	11	46	L1365V	c.4093T>G	10	47

**28**	**Ovarian**	K339_L347delinsR	c.1016_1040delinsG	10	[illegible]	K339fs	c.1016delA	10	25
**29**	**Ovarian**	T276fs	c.815_824dup10	10	[illegible]	A254_V271del	c.761_814del54	10	14
**30**	**Ovarian**	D430_I441delinsRLTGQ	c.1288_1323delins15	10	[illegible]	D430fs	c.1287dupA	10	51
**31**	**Pancreatic**	Q1756_R1758delinsPG	c.5267_5272delinsCAG	19	[illegible]	Q1756fs	c.5266dupC	19	50
**32**	**Pancreatic**	E1250_S1262del	c.3747_3785del39	10	[illegible]	E1250*	c.3748G>T	10	66

		***BRCA2* mutation 1**				***BRCA2* mutation 2**				***BRCA2* mutation 3**			
	**Cancer Type**	**p. changes**	**c. changes**	**Exon**	**VAF**	**p. changes**	**c. changes**	**Exon**	**VAF**	**p. changes**	**c. changes**	**Exon**	**VAF**
**33**	**Bladder**	I1859fs	c.5576_5579delTTAA	11	69	I1859del	c.5576_5578delTTA	11	18	S646_P655delinsT	c.1937_1963del27	11	18
**34**	**Breast**	Q2157_K2162delinsISTR	c.6468_6485delins12	11	40	K2162fs	c.6486_6489delACAA	11	36				
**35**	**Breast**	F2011_V2014del	c.6030_6041del12	11	19	K2013*	c.6037A>T	11	76				
**36**	**Breast**	D935_Y949del	c.2804_2848del45	11	68	A938fs	c.2808_2811delACAA	11	14				
**37**	**Breast**	K1881fs	c.5641_5644delAAAT	11	14	S1882_K1883del	c.5644_5649delTCAAAA	11	13	Y3098*	c.9294C>G	25	49
**38**	**Breast**	N243fs	c.729delT	9	7	c.7618-1_7625delins8	c.7618-1_7625delins8	16	7	c.7618-1G>A	c.7618-1G>A	16	40
**39**	**Breast**	L105S	c.314T>C	3	11	L105*	c.314T>G	3	38				
**40**	**Breast**	K1057fs	c.3170_3174delAGAAA	11	55	K1057_Q1063delinsTEQA	c.3170_3189delins11	11	29				
**41**	**Breast**	R2520S	c.7558_7559delinsTC	15	63	R2520*	c.7558C>T	15	18				
**42**	**Breast**	E425_K426del	c.1275_1280delAAAAGA	10	27	D427fs	c.1278delA	10	49				
**43**	**Breast**	N422fs	c.1265delA	10	37	N422_K436delinsIFQKKTYYTENKRQ	c.1265_1306delins39	10	33				
**44**	**Breast**	L2015_N2030delinsTNIQTSSQEKKI	c.6043_6090delins36	11	30	D2023_N2030delinsQEKKI	c.6067_6090delins15	11	9	T2031fs	c.6091dupA	11	34
**45**	**Breast**	M1300_E1311del	c.3899_3934del36	11	28	G1303_T1310del	c.3908_3931del24	11	18	E1308*	c.3922G>T	11	24
**46**	**Breast**	V1775_Q1782delinsLRMLKI	c.5323_5346delins18	11	19	N1784fs	c.5350_5351delAA	11	37				
**47**	**Breast**	K1881fs	c.5641_5644delAAAT	11	22	K1881_E1895delinsQKFAKRKLWQ	c.5641_5683delins28	11	45				
**48**	**Breast**	F2130I	c.6388T>A	11	13	N2189fs	c.6566dupA	11	71	N2189_C2212delinsKR	c.6567_6634delinsAC	11	17
**49**	**Breast**	E83_L88delinsDW	c.249_263delinsTTG	3	18	Q84*	c.250C>T	3	42				
**50**	**Breast**	Q649Y	c.1945_1947delinsTAC	11	59	Q649*	c.1945C>T	11	17	D750N	c.2248G>A	11	67
**51**	**Breast**	S646_P655delinsT	c.1937_1963del27	11	18	P655fs	c.1964delC	11	35				
**52**	**Breast**	V2102_L2136delinsLNYQI	c.6304_6406delins13	11	22	N2135fs	c.6405_6409delCTTAA	11	65				
**53**	**Breast**	C1975_S1982delinsSK	c.5922_5946delins7	11	53	S1982fs	c.5946delT	11	23				
**54**	**Breast**	S646_P655delinsT	c.1937_1963del27	11	18	P655fs	c.1964delC	11	46				

**55**	**Cancer of Unknown Primary**	Q699Y	c.2095_2097delinsTAT	11	5	Q699*	c.2095C>T	11	62				
**56**	**Cholangiocarcinoma**	E97*	c.289G>T	3	14	W1692fs	c.5073dupA	11	15	C1820R	c.5458_5460delinsAGA	11	9
**57**	**Malignant Pleural Mesothelioma**	N2208_T2214delinsSFVLP	c.6623_6640delins12	11	25	Y2215fs	c.6641dupC	11	60				
**58**	**Neuroendocrine tumors**	K1850_F1866del	c.5547_5598delinsA	11	79	I1859fs	c.5576_5579delTTAA	11	9				
**59**	**Ovarian**	N1973_S1989del	c.5917_5967del51	11	11	S1982fs	c.5946delT	11	51				
**60**	**Ovarian**	C1853_I1859delinsLN	c.5558_5577delinsTAAAC	11	57	I1859fs	c.5576_5579delTTAA	11	21				
**61**	**Ovarian**	E471K	c.1411G>A	10	43	E471*	c.1411G>T	10	51				
**62**	**Ovarian**	E1311_Y1313delinsST	c.3931_3939delinsTCAACA	11	7	E1311*	c.3931G>T	11	30	K3059N	c.9177A>C	24	31
**63**	**Ovarian**	V1283_S1284del	c.3848_3853delTAAGTG	11	31	S1284fs	c.3849_3852delAAGT	11	39				
**64**	**Ovarian**	S1979_V1999delinsM	c.5936_5995del60	11	35	S1982fs	c.5946delT	11	33				
**65**	**Ovarian**	H1918Y	c.5752C>T	11	76								
**66**	**Ovarian**	R3128W	c.9382_9384delinsTGG	25	77	R3128*	c.9382C>T	25	11				
**67**	**Prostate**	Y1894K	c.5680_5682delinsAAG	11	52	Y1894*	c.5682C>G	11	21				
**68**	**Prostate**	S1982fs	c.5946delT	11	57	S1982_V1999delinsRENLSRYQM	c.5946_5995delins23	11	27				
**69**	**Prostate**	R2520S	c.7558_7559delinsTC	15	15	R2520*	c.7558C>T	15	40				
**70**	**Prostate**	A1972_S1985del	c.5915_5956del42	11	11	S1982fs	c.5946delT	11	70				
**71**	**Prostate**	D1484_P1496del	c.4451_4489del39	11	80	H1488fs	c.4464_4465delCA	11	9				
**72**	**Endometrial**	Q66Y	c.196_198delinsTAT	3	14	Q66*	c.196C>T	3	53				
**73**	**Endometrial**	N863fs	c.2589delinsAA	11	26	N863K	c.2589T>A	11	20	I1607V	c.4819A>G	11	52
**74**	**Endometrial**	F12fs	c.36delT	2	53	F12_K21delinsLKFLRH	c.36_63delins16	2	23				
**75**	**Endometrial**	D1280_S1284del	c.3840_3854del15	11	60	V1283fs	c.3847_3848delGT	11	20				

Table S2. Gene lists on the 592-gene panel and WES panel with high coverage.

**592 gene panel**	WES panel high coverage genes
ABI1	ABCB11
ABL1	ABL1
ABL2	ABL2
ACKR3	ABRAXAS1
ACSL3	ACD
ACSL6	ACKR3
ADGRA2	ACSL3
AFDN	ACSL6
AFF1	ACVR1
AFF3	ACVR1B
AFF4	ADGRA2
AKAP9	AFDN
AKT1	AFF1

AKT2	AFF3
AKT3	AIP
ALDH2	AJUBA
ALK	AKT1
AMER1	AKT2
APC	AKT3
AR	ALK
ARAF	ALOX12B
ARFRP1	AMER1
ARHGAP26	ANKRD26
ARHGEF12	APC
ARID1A	APLNR
ARID2	AR
ARNT	ARAF
ASPSCR1	ARFRP1
ASXL1	ARHGAP35
ATF1	ARHGEF12
ATIC	ARID1A
ATM	ARID1B
ATP1A1	ARID2
ATP2B3	ARID5B
ATR	ARNT
ATRX	ASPSCR1
AURKA	ASXL1
AURKB	ASXL2
AXIN1	ATF1
AXL	ATIC
BAP1	ATM
BARD1	ATP1A1
BCL10	ATR
BCL11A	ATRX
BCL11B	AURKA
BCL2	AURKB
BCL2L11	AXIN1
BCL2L2	AXIN2
BCL3	AXL
BCL6	B2M
BCL7A	BAP1
BCL9	BARD1
BCOR	BCL10
BCORL1	BCL11A
BCR	BCL11B
BIRC3	BCL2
BLM	BCL2L1
BMPR1A	BCL2L11
BRAF	BCL2L12
BRCA1	BCL2L2
BRCA2	BCL3
BRD3	BCL6
BRD4	BCL9

BRIP1	BCOR
BTG1	BCORL1
BTK	BIRC3
BUB1B	BLM
C15orf65	BMPR1A
CACNA1D	BRAF
CALR	BRCA1
CAMTA1	BRCA2
CANT1	BRD3
CARD11	BRD4
CARS	BRIP1
CASP8	BTG1
CBFA2T3	BTG2
CBFB	BTK
CBL	BUB1B
CBLB	C15orf65
CBLC	CACNA1D
CCDC6	CALR
CCNB1IP1	CAMTA1
CCND1	CARD11
CCND2	CARS
CCND3	CASP8
CCNE1	CBFA2T3
CD274	CBFB
CD74	CBL
CD79A	CBLB
CD79B	CCDC6
CDC73	CCND1
CDH1	CCND2
CDH11	CCND3
CDK12	CCNE1
CDK4	CD22
CDK6	CD274
CDK8	CD70
CDKN1B	CD74
CDKN2A	CD79A
CDKN2B	CD79B
CDKN2C	CDC73
CDX2	CDH1
CEBPA	CDH11
CHCHD7	CDH23
CHEK1	CDK12
CHEK2	CDK4
CHIC2	CDK6
CHN1	CDK8
CIC	CDKN1A
CIITA	CDKN1B
CLP1	CDKN1C
CLTC	CDKN2A
CLTCL1	CDKN2B

CNBP	CDKN2C
CNOT3	CDX2
CNTRL	CEBPA
COL1A1	CHD2
COPB1	CHD4
COX6C	CHEK1
CREB1	CHEK2
CREB3L1	CHIC2
CREB3L2	CHN1
CREBBP	CIC
CRKL	CIITA
CRLF2	CLTCL1
CRTC1	CLYBL
CRTC3	CNBP
CSF1R	CNOT3
CSF3R	COX6C
CTCF	CREB1
CTLA4	CREB3L1
CTNNA1	CREB3L2
CTNNB1	CREBBP
CYLD	CRKL
CYP2D6	CRLF2
DAXX	CRTC1
DDB2	CRTC3
DDIT3	CSF1R
DDR2	CSF3R
DDX10	CTCF
DDX5	CTLA4
DDX6	CTNNA1
DEK	CTNNB1
DICER1	CUL3
DNM2	CUL4A
DNMT3A	CUX1
DOT1L	CXCR4
EBF1	CYLD
ECT2L	CYP17A1
EGFR	CYP2D6
EIF4A2	DAXX
ELF4	DDB2
ELK4	DDIT3
ELL	DDR1
ELN	DDR2
EML4	DDX3X
EMSY	DDX41
EP300	DDX6
EPHA3	DEK
EPHA5	DICER1
EPHB1	DIS3
EPS15	DIS3L2
ERBB2	DKC1

ERBB3	DMC1
ERBB4	DNA2
ERC1	DNAJB1
ERCC1	DNMT3A
ERCC2	DOT1L
ERCC3	EBF1
ERCC4	ECT2L
ERCC5	EED
ERG	EGFR
ESR1	EGLN1
ETV1	EIF1AX
ETV4	EIF4A2
ETV5	ELF3
ETV6	ELK4
EWSR1	ELOC
EXT1	EME1
EXT2	EME2
EZH2	EML4
EZR	EMSY
FAM46C	EP300
FANCA	EPCAM
FANCC	EPHA2
FANCD2	EPHA3
FANCE	EPHA5
FANCF	EPHA7
FANCG	EPHB1
FANCL	EPHB4
FAS	ERBB2
FBXO11	ERBB3
FBXW7	ERBB4
FCRL4	ERC1
FEV	ERCC1
FGF10	ERCC2
FGF14	ERCC3
FGF19	ERCC4
FGF23	ERCC5
FGF3	ERCC6
FGF4	EREG
FGF6	ERG
FGFR1	ERRFI1
FGFR1OP	ESR1
FGFR2	ETS1
FGFR3	ETV1
FGFR4	ETV4
FH	ETV5
FHIT	ETV6
FIP1L1	EWSR1
FLCN	EXO1
FLI1	EXT1
FLT1	EXT2

FLT3	EZH2
FLT4	EZR
FNBP1	FAM46C
FOXA1	FANCA
FOXL2	FANCB
FOXO1	FANCC
FOXO3	FANCD2
FOXO4	FANCE
FOXP1	FANCF
FSTL3	FANCG
FUBP1	FANCI
FUS	FANCL
GAS7	FANCM
GATA1	FAS
GATA2	FAT1
GATA3	FAT3
GID4	FBXO11
GMPS	FBXW7
GNA11	FCRL4
GNA13	FEN1
GNAQ	FGF10
GNAS	FGF12
GOLGA5	FGF14
GOPC	FGF19
GPC3	FGF23
GPHN	FGF3
GRIN2A	FGF4
GSK3B	FGF6
H3F3A	FGFR1
H3F3B	FGFR1OP
HERPUD1	FGFR2
HEY1	FGFR3
HGF	FGFR4
HIP1	FH
HIST1H3B	FHIT
HIST1H4I	FIP1L1
HLF	FLCN
HMGA1	FLI1
HMGA2	FLT1
HMGN2P46	FLT3
HNF1A	FLT4
HNRNPA2B1	FNBP1
HOOK3	FOXA1
HOXA11	FOXL2
HOXA13	FOXO1
HOXA9	FOXO3
HOXC11	FOXO4
HOXC13	FOXP1
HOXD11	FRS2
HOXD13	FSTL3

HRAS	FUBP1
HSP90AA1	FUS
HSP90AB1	FYN
IDH1	GABRA6
IDH2	GALNT12
IGF1R	GATA1
IKBKE	GATA2
IKZF1	GATA3
IL2	GATA4
IL21R	GATA6
IL6ST	GEN1
IL7R	GID4
INHBA	GLI1
IRF4	GLI2
IRS2	GMPS
ITK	GNA11
JAK1	GNA13
JAK2	GNAQ
JAK3	GNAS
JAZF1	GOPC
JUN	GPC3
KAT6A	GPS2
KAT6B	GREM1
KCNJ5	GRIN2A
KDM5A	GRM3
KDM5C	GSK3B
KDM6A	H2AFX
KDR	H3F3A
KDSR	H3F3B
KEAP1	HDAC1
KIAA1549	HEY1
KIF5B	HGF
KIT	HIF1A
KLF4	HIST1H3B
KLHL6	HIST1H3C
KLK2	HIST1H4I
KMT2A	HLF
KMT2C	HMGA2
KMT2D	HMGN2P46
KNL1	HNF1A
KRAS	HOOK3
KTN1	HOXA11
LASP1	HOXA13
LCK	HOXA9
LCP1	HOXB13
LGR5	HOXD13
LHFPL6	HRAS
LIFR	HRG
LMO1	HRR
LMO2	HSD3B1

LPP	HSP90AA1
LRIG3	HSP90AB1
LRP1B	ID2
LYL1	ID3
MAF	IDH1
MAFB	IDH2
MALT1	IFNGR1
MAML2	IGF1R
MAP2K1	IGF2
MAP2K2	IKBKE
MAP2K4	IKZF1
MAP3K1	IL7R
MAX	INHBA
MCL1	INPP4B
MDM2	IRF1
MDM4	IRF2
MDS2	IRF4
MECOM	IRS2
MED12	ITK
MEF2B	JAK1
MEN1	JAK2
MET	JAK3
MITF	JAZF1
MKL1	JUN
MLF1	KAT6A
MLH1	KAT6B
MLLT1	KCNJ5
MLLT10	KDM5A
MLLT11	KDM5C
MLLT3	KDM6A
MLLT6	KDR
MN1	KDSR
MNX1	KEAP1
MPL	KEL
MRE11	KIAA1549
MSH2	KIF1B
MSH6	KIF5B
MSI2	KIT
MSN	KLF4
MTCP1	KLHL6
MTOR	KLK2
MUC1	KMT2A
MUTYH	KMT2C
MYB	KMT2D
MYC	KRAS
MYCL	LCK
MYCN	LDLR
MYD88	LHFPL6
MYH11	LIFR
MYH9	LIG1

NACA	LMNA
NBN	LMO1
NCKIPSD	LMO2
NCOA1	LOH
NCOA2	LPP
NCOA4	LRP1B
NDRG1	LTK
NF1	LYN
NF2	LZTR1
NFE2L2	MAF
NFIB	MAFB
NFKB2	MAGI2
NFKBIA	MALT1
NIN	MAML2
NKX2-1	MAP2K1
NONO	MAP2K2
NOTCH1	MAP2K4
NOTCH2	MAP3K1
NPM1	MAP3K13
NR4A3	MAPK1
NRAS	MAPK3
NSD1	MAX
NSD2	MBD4
NSD3	MCL1
NT5C2	MDC1
NTRK1	MDM2
NTRK2	MDM4
NTRK3	MDS2
NUMA1	MECOM
NUP214	MED12
NUP93	MEF2B
NUP98	MEN1
NUTM1	MERTK
NUTM2B	MET
OLIG2	MGA
OMD	MGMT
P2RY8	MITF
PAFAH1B2	MKNK1
PAK3	MLF1
PALB2	MLH1
PATZ1	MLH3
PAX3	MLLT10
PAX5	MLLT11
PAX7	MLLT3
PAX8	MN1
PBRM1	MPL
PBX1	MRE11
PCM1	MSH2
PCSK7	MSH3
PDCD1	MSH6

PDCD1LG2	MSI2
PDE4DIP	MST1R
PDGFB	MTAP
PDGFRA	MTCP1
PDGFRB	MTOR
PDK1	MUC1
PER1	MUS81
PHF6	MUTYH
PHOX2B	MYB
PICALM	MYC
PIK3CA	MYCL
PIK3CG	MYCN
PIK3R1	MYD88
PIK3R2	MYH11
PIM1	MYH9
PLAG1	NBN
PML	NCOA2
PMS1	NCOA3
PMS2	NCOA4
POLE	NCOR1
POT1	NDRG1
POU2AF1	NF1
POU5F1	NF2
PPARG	NFE2L2
PPP2R1A	NFIB
PRCC	NFKB2
PRDM1	NFKBIA
PRDM16	NFKBIE
PRF1	NIN
PRKAR1A	NKX2-1
PRKDC	NONO
PRRX1	NOTCH1
PSIP1	NOTCH2
PTCH1	NOTCH3
PTEN	NPM1
PTPN11	NR4A3
PTPRC	NRAS
RABEP1	NSD1
RAC1	NSD2
RAD21	NSD3
RAD50	NT5C2
RAD51	NTHL1
RAD51B	NTRK1
RAF1	NTRK2
RALGDS	NTRK3
RANBP17	NUP214
RAP1GDS1	NUP93
RARA	NUP98
RB1	NUTM1
RBM15	OLIG2

RECQL4	P2RY8
REL	PAFAH1B2
RET	PAK1
RHOH	PAK3
RICTOR	PALB2
RMI2	PARP1
RNF213	PARP2
RNF43	PARP3
ROS1	PAX3
RPL10	PAX5
RPL22	PAX7
RPL5	PAX8
RPN1	PBRM1
RPTOR	PBX1
RUNX1	PCM1
RUNx1T1	PDCD1
SBDS	PDCD1LG2
SDC4	PDE4DIP
SDHAF2	PDGFB
SDHB	PDGFRA
SDHC	PDGFRB
SDHD	PDK1
SEPT5	PER1
SEPT6	PHF6
SEPT9	PHOX2B
SET	PIK3C2B
SETBP1	PIK3C2G
SETD2	PIK3CA
SF3B1	PIK3CB
SFPQ	PIK3CD
SH2B3	PIK3CG
SH3GL1	PIK3R1
SLC34A2	PIK3R2
SLC45A3	PIM1
SMAD2	PLAG1
SMAD4	PLCG2
SMARCA4	PML
SMARCB1	PMS1
SMARCE1	PMS2
SMO	POLD1
SNX29	POLD2
SOCS1	POLD3
SOX10	POLD4
SOX2	POLE
SPECC1	POLH
SPEN	POLQ
SPOP	POT1
SRC	POU2AF1
SRGAP3	PPARG
SRSF2	PPM1D

SRSF3	PPP2R1A
SS18	PPP2R2A
SS18L1	PPP6C
SSX1	PRCC
STAG2	PRDM1
STAT3	PREX2
STAT4	PRF1
STAT5B	PRKACA
STIL	PRKAR1A
STK11	PRKCH
SUFU	PRKCI
SUZ12	PRKDC
SYK	PRKN
TAF15	PRRX1
TAL1	PRSS8
TAL2	PTCH1
TBL1XR1	PTCH2
TCEA1	PTEN
TCF12	PTK2B
TCF3	PTPN11
TCF7L2	PTPN22
TCL1A	PTPRC
TERT	PTPRD
TET1	PTPRO
TET2	PTPRT
TFE3	QKI
TFEB	RABL3
TFG	RAC1
TFPT	RAD21
TFRC	RAD50
TGFBR2	RAD51
THRAP3	RAD51B
TLX1	RAD51C
TLX3	RAD51D
TMPRSS2	RAD52
TNFAIP3	RAD54B
TNFRSF14	RAD54L
TNFRSF17	RAF1
TOP1	RANBP2
TP53	RARA
TPM3	RASA1
TPM4	RB1
TPR	RBBP8
TRAF7	RBM10
TRIM26	RCAN1
TRIM27	RECQL4
TRIM33	REL
TRIP11	REL
TRRAP	RELA
TSC1	RET

TSC2	RHEB
TSHR	RHOA
TTL	RHOH
U2AF1	RICTOR
UBR5	RINT1
USP6	RIT1
VEGFA	RMI2
VEGFB	RNF43
VHL	ROS1
VTI1A	RPA1
WAS	RPA2
WDCP	RPA3
WIF1	RPA4
WISP3	RPL10
WRN	RPL22
WT1	RPL5
WWTR1	RPN1
XPA	RPTOR
XPC	RRAS2
XPO1	RSPO1
YWHAE	RSPO2
ZBTB16	RSPO3
ZMYM2	RUNX1
ZNF217	RUNx1T1
ZNF331	SBDS
ZNF384	SDC4
ZNF521	SDHA
ZNF703	SDHAF2
ZRSR2	SDHB
	SDHC
	SDHD
	SEM1
	SERPINB3
	SET
	SETBP1
	SETD2
	SF3B1
	SFPQ
	SGK1
	SH2B3
	SLC34A2
	SLIT2
	SLX4
	SMAD2
	SMAD3
	SMAD4
	SMARCA1
	SMARCA4
	SMARCB1
	SMARCE1

	SMC3
	SMO
	SNCAIP
	SNX29
	SOCS1
	SOS1
	SOX10
	SOX2
	SOX9
	SPECC1
	SPEN
	SPOP
	SPRED1
	SPTA1
	SRC
	SRGAP3
	SRSF2
	SRSF3
	SS18
	SSBP1
	STAG2
	STAT3
	STAT4
	STAT5B
	STAT6
	STIL
	STK11
	SUFU
	SUZ12
	SYK
	TAF1
	TAF15
	TAL1
	TAL2
	TBX3
	TBXT
	TCEA1
	TCF3
	TCF7L2
	TEK
	TERC
	TERF2IP
	TERT
	TET1
	TET2
	TFE3
	TFEB
	TFG
	TFRC
	TGFBR1

TGFBR2
THRAP3
TIPARP
TLX3
TMEM127
TNF
TNFAIP3
TNFRSF14
TOP1
TOP2A
TOP3A
TOP3B
TP53
TPM3
TPM4
TRAF3
TRAF7
TRIM27
TRRAP
TSC1
TSC2
TSHR
TSHZ3
TYK2
TYRO3
U2AF1
UBE2T
UBR5
USP6
VEGFA
VHL
VTI1A
WAS
WDCP
WISP3
WRN
WT1
WWTR1
XPA
XPC
XPO1
XRCC1
XRCC2
XRCC3
YAP1
YES1
YWHAE
ZBTB16
ZBTB2
ZFHX3

	ZNF217
	ZNF331
	ZNF384
	ZNF521
	ZNF703
	ZNRF3
	ZRSR2
